# A Late Cretaceous dinosaur and crocodyliform faunal association–based on isolate teeth and osteoderms–at Cerro Fortaleza Formation (Campanian-Maastrichtian) type locality, Santa Cruz, Argentina

**DOI:** 10.1371/journal.pone.0256233

**Published:** 2021-09-08

**Authors:** Ariana Paulina-Carabajal, Francisco T. Barrios, Ariel H. Méndez, Ignacio A. Cerda, Yuong-Nam Lee

**Affiliations:** 1 Instituto de Investigaciones en Biodiversidad y Medioambiente (CONICET-Universidad Nacional del Comahue), San Carlos de Bariloche, Río Negro, Argentina; 2 Museo Provincial de Ciencias Naturales “Profesor Olsacher”, Zapala, Neuquén, Argentina; 3 Instituto Patagónico de Geología y Paleontología (CCT CONICET-CENPAT), Puerto Madryn, Chubut, Argentina; 4 Instituto de Investigación en Paleobiología y Geología (CONICET- Universidad Nacional de Río Negro), Museo Carlos Ameghino, Cipolletti, Río Negro, Argentina; 5 School of Earth and Environmental Sciences, Seoul National University, Seoul, Republic of Korea; Universidade de Sao Paulo, BRAZIL

## Abstract

The Late Cretaceous dinosaur record in southern South America has been improved recently; particularly with findings from Chorrillo and Cerro Fortaleza formations, both bearing ankylosaur remains, a clade that was not previously recorded in the Austral Basin. The dinosaur fauna of the type locality of Cerro Fortaleza Formation is known from -and biased to- large-sized sauropod remains and a single described taxon, the titanosaur *Dreadnoughtus schrani*. Here, we report the taxonomic composition of a site preserving thirteen isolated teeth and several osteoderms belonging to three dinosaur clades (Abelisauridae, Titanosauria, and Nodosauridae), and at least one clade of notosuchian crocodyliforms (Peirosauridae). They come from sediments positioned at the mid-section of the Cerro Fortaleza Formation, which is Campanian-Maastrichtian in age, adding valuable information to the abundance and biodiversity of this Cretaceous ecosystem. Since non-titanosaur dinosaur bones are almost absent in the locality, the teeth presented here provide a window onto the archosaur biodiversity of the Late Cretaceous in southern Patagonia. The nodosaurid tooth and small armor ossicles represent the first record of ankylosaurs for this stratigraphic unit. The peirosaurid material also represents the most austral record of the clade in South America.

## Introduction

**Fig 1:**
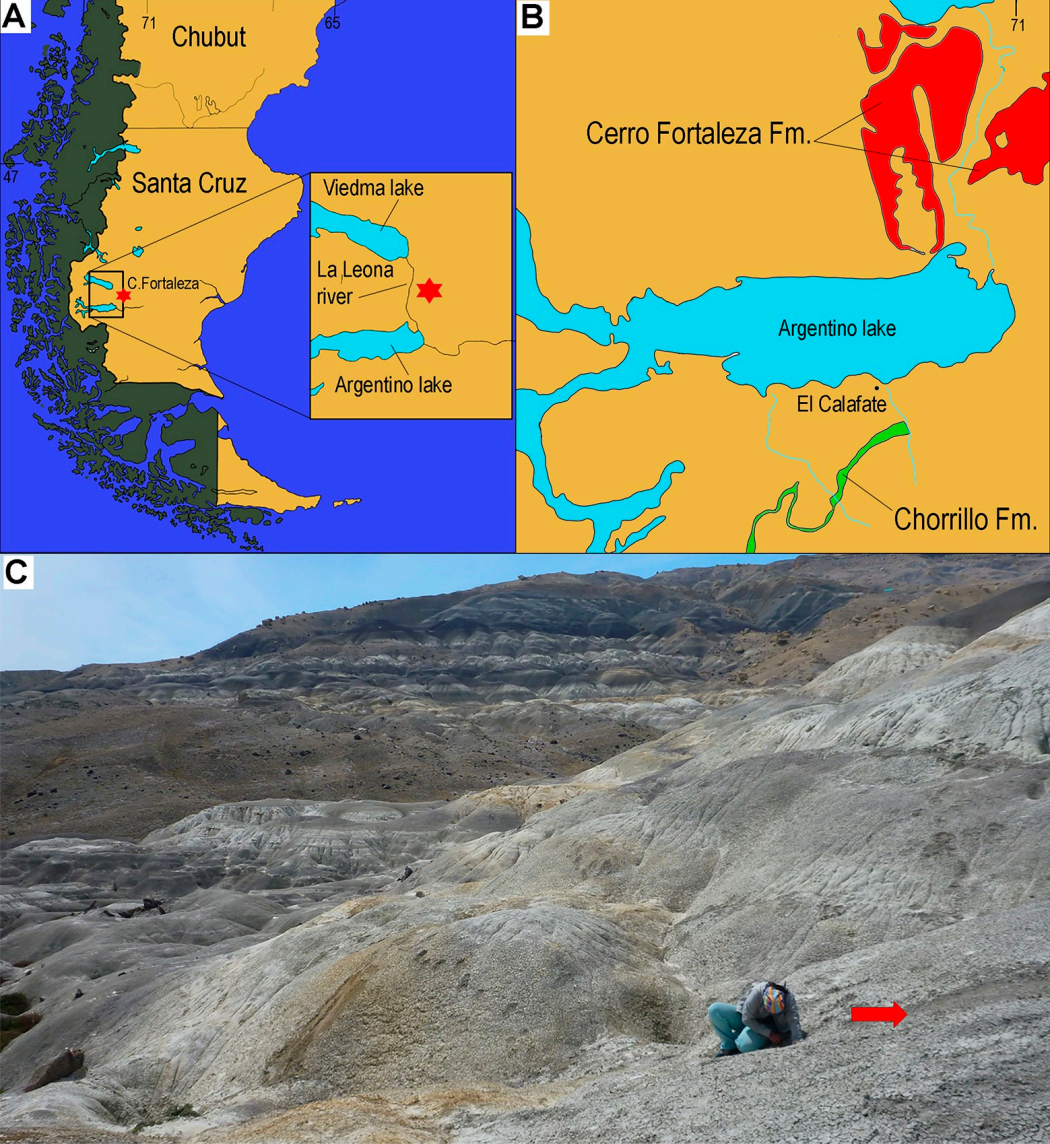
Location map showing the provenance of the teeth and osteoderms. Southern South America, with the Cerro Fortaleza type-locality indicated by the star (A). Region between Viedma and Argentino lakes showing the Cerro Fortaleza Formation (red color) outcropping at both sides of La Leona river. The Chorrillo Formation is indicated in green (B). Photography of the site, the red arrow indicates the level with teeth and osteoderms (C). B is based on [2,16].

The Cerro Fortaleza Formation (Campanian-Maastrichtian in age) crops out along the West and East margins of La Leona River, and south of Lago Viedma, Santa Cruz Province, Argentina (Fig 1). This formation is part of the Austral-Magallanes Basin, a sedimentary infill accumulated during the Late Cretaceous dominated by deep-marine and coastal deposits, located at the southern edge of the South American plate. The continental sedimentary succession within the basin, however, remains poorly known and different lithostratigraphic schemes entered the literature [1,2]. The type locality of Cerro Fortaleza Formation is exposed along the oriental margin of La Leona river at the homonymous hill (Cerro Fortaleza), where its largest exposure–approximately 400 m–is observed [1, and references therein]. The geology and stratigraphy of this area have been thoroughly studied along the last century, but the interpretations about the exposed rocks remain controversial [1–4]. For example, the Cerro Fortaleza locality and near areas were given contradicting ages in the literature (from Cenomanian to Campanian-Maastrichtian), and other formation names were proposed for this locality, such as “Mata Amarilla” (Cenomanian beds now considered to outcrop only at areas near Tres Lagos city), or “Pari Aike” [e.g. 3,5–8]. A recent sedimentological analysis [2] included the Cerro Fortaleza, La Anita, La Irene, and Chorrillo (another dinosaur-bearing unit [9,10]) formations as lithologically similar beds under the denomination “Uppermost Cretaceous Continental Deposits”. Regardless of their names, the dinosaur-bearing units were generally considered as Campanian-Maastrichtian [4,6,11–16].

The dinosaur taxa described for Cerro Fortaleza Formation include the theropods *Orkoraptor burkei* [6] and *Austrocheirus isasii* [12], the elasmarian ornithopod *Talenkauen santacrucensis* [13], and the large-sized titanosaurids *Puertasaurus reuili* [14] and *Dreadnoughtus schrani* [11]. Whereas most taxa were found at the south of Lago Viedma, west of La Leona river, *D*. *schrani* is the only species found at Cerro Fortaleza locality up to date (Fig 1A and 1B). In this locality, the most commonly found dinosaur remains are biased to large-sized sauropod bones, as in other outcroppings of the Cerro Fortaleza Formation [15], and only an abelisaurid theropod metatarsus has been reported so far [17]. As for crocodyliforms, their record in the unit come from the area west of La Leona river and corresponds to an indeterminate scute [15].

During a fieldwork carried out in December 2016 at the Cerro Fortaleza locality, several isolate dinosaur and crocodyliform teeth were recovered from the Cerro Fortaleza Formation (Fig 1C). The sample presented here is not large, and most specimens consist of fractured and/or highly eroded dinosaur and crocodyliform teeth (MPM-PV-18805.1–14). However, the association observed in the sample is rich in terms of archosaur taxonomic diversity for the Austral Basin; particularly, peirosaurid teeth are reported here for the first time. Interestingly, the other highly diverse dinosaur-bearing unit in the basin, the Chorrillo Formation, located 160 km south from the Cerro Fortaleza locality, has been recently considered as late Campanian-early Maastrichtian based on its faunal content [9,10]. The correlation between the two formations is not clear, and there are differences in the faunas documented in the Chorrillo and the probably underlying Cerro Fortaleza formations. Both units generally share the same dinosaur clades [9,10], i.e., titanosaurs, theropods (abelisauroids, megaraptorids), elasmarian ornithopods, and ankylosaurs. Taxonomical differences are at generic level, and one group of non-avian dinosaurs–hadrosaurids–have been documented only in the Chorrillo Formation.

In this context, isolated teeth are key pieces of evidence to assess vertebrate paleo-ecological diversity [18]. Tooth measurements were first employed by Currie et al. [19], and Farlow et al. [20] for systematic identification of theropod teeth, and later authors followed or modified this method to document similar isolated remains [e.g. 21,22]. More recently, a number of workers have successfully used dinosaur tooth morphology for taxonomic purposes [18,22,23]. The aim of this study is to describe the morphology of the isolated dinosaur and crocodyliform teeth from a Late Cretaceous microremains site. The vertebrate fossils from the Cerro Fortaleza Formation, particularly the ankylosaur (probable nodosaurid) specimen, may help to increase our understanding about the vertebrate paleoecology, paleoenvironments, and possible biotic dispersal events during the Late Cretaceous from the southern latitudes.

## Materials and methods

A batch of 13 teeth and 9 osteoderms are under the collection number MPM-PV-18805, Museo Padre Molina (Río Gallegos, Santa Cruz, Argentina). The teeth (MPM-PV-18805.1–13) were found isolated, and all correspond to dinosaur and crocodyliform crowns missing the roots. The osteoderms (MPM-PV-18805.14–22) correspond to small sized ankylosaur interstitial ossicles. All the specimens were collected from the surface in an area not larger than 4 m^2^ (GPS coordinates are -49.94°, -72.05°). The location of the site corresponds approximately to the mid-to-upper portions of the Cerro Fortaleza Formation type-section, interpreted as sediments deposited in fluvial-tidal environments [8,24,25].

The terminology used to describe the theropod teeth follows Hendrickx et al. [18,23], and the crocodile teeth follows Legasa et al. [26–28] and Ösi [29]. The following dental measurements and ratios (proposed by Smith et al. [21] and updated by Hendrickx et al [18]) were used in the descriptions (Table 1).

**Table 1:** Cerro Fortaleza theropod and crocodyliform teeth measurements (in mm).

	CH	CBL	CBW	CBR	DC
**Theropoda**					
**MPM-PV-18805.1**	13+	9	4	0.44	11.5
**Peirosaurid**					
**MPM-PV-18805.5**	25.0	11.0	9.11	0.83	2.8
**MPM-PV-18805.6**	18.3	10.0	7.63	0.76	3.0
**MPM-PV-18805.7**	6.76	6.23	4.28	0.69	4.2
**MPM-PV-18805.8**	6.36	5.63	4.08	0.73	3.5
**MPM-PV-18805.9**	4.86	3.35**	3.61	-	4.0
**MPM-PV-18805.10**	3.66	4.42**	3.47	0.79**	4.8
**MPM-PV-18805.11**	6.17	5.64	4.19	0.74	3.4
**MPM-PV-18805.12**	8.76	-	5.19**	-	5.5
**MPM-PV-18805.13**	6.79**	5.09	4.45	0.87	5.5

Abbreviations: CBL, crown base length measured at the base of the crown from its mesialmost to distalmost extention (excluding the carinae); CBR, crown base ratio, numeral value derived from dividing CBW and CBL (= labiolingual compression); CBW, crown base width, labiolingual extension of the crown at its base; CH, crown height; DC, denticle density (per 5 mm in theropods; per 1 mm in crocodyliforms).

*at mid-crown.

**partial measurement.

Histological thin sections from two dermal ossicles (MPM-PV-18805.19 and 18805.22) were prepared at the Carlos Ameghino Museum (Cipolletti, Río Negro Province, Argentina). The slices were prepared using standard methods outlined by Cerda et al. [30] and studied using a petrographic polarizing microscope (Leica DM 750P). The nomenclature and definitions of structures used in this study are derived from Francillon-Vieillot et al. [31] and Cerda et al. [32].

Geological settings. The sandstones and mudstones of Cerro Fortaleza Formation (maximum exposure of 390m) were deposited in paralic, fluvial, and fluvial-tidal environments [1]. This can explain the presence of fish scales in the sample, which may correspond to both freshwater or marine forms.

## Fossil assemblage and taxonomic affinities

### Theropod remains

Theropoda Marsh, 1881

Abelisauroidea Bonaparte and Novas, 1985

#### Abelisauroidea indet

**Fig 2:**
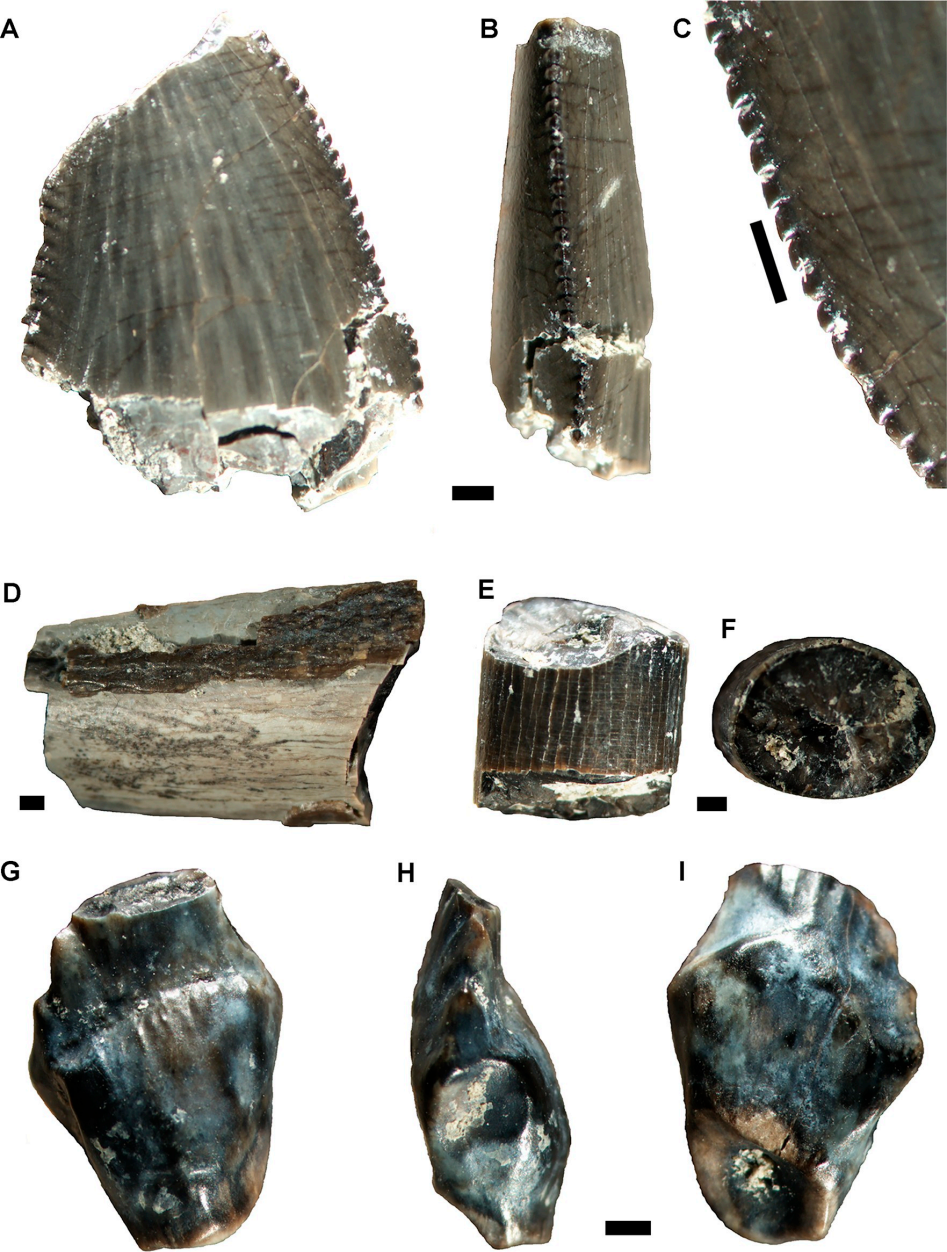
Dinosaur teeth. A-C, Abelisauria indet. (MPM-PV-18805.1), partial tooth in lingual or labial (A) and mesial (B) views; detail of mesial denticles (C). D, partial tooth (MPM-PV-18805.2) of a non-identified archosaur, preserving a patch of enamel showing a highly marked ornamentation. E-F, Titanosauria indet. (MPM-PV-18805.3), partial tooth. G-I, Ankylosaur (MPM-PV-18805.4) partial tooth in labial (G), distal (H) and lingual (I) views. Scale bars = 1 mm (except in A, B = 5mm).

MPM-Pv-18805.1 (Fig 2A–2C) is an incomplete ziphodont tooth (preserved length = 11 mm, if the crown was complete it could have reached 19–20 mm), missing the crown apex and the root. In lateral view, the crown is posteriorly curved, with slightly convex mesial and straighter distal margins. The tooth is strongly labio-lingually narrow (CBR = 0.44; labiolingual width is 44.4% of mesiodistal length), and the cross-section type is lenticular or D-shaped. In mesial/distal views, the serrated mesial and distal carinae are straight and centrally positioned on the crown (Fig 2A and 2B). The enamel is smooth (corresponds to the irregular type described by [18]). This irregular, smooth enamel texture is observed in abelisaurid teeth [18,22,33]. Denticles on the mesial carina have asymmetrical convex margins, are hooked and strongly apically recurved. They show separate narrow interdenticular spaces (interdenticular slit in [18]). Distal denticles are proximo-distally longer (proximodistally subrectangular denticles) than mesial denticles, and slightly apically oriented or chisel-like. Denticle density is approximately the same in both carinae, e.g., number of denticles per 5 mm at the mid-crown on the mesial carina is 11.5 (Fig 2C), and 11.3 on the distal carina (Table 1).

Abelisaurid tooth traits defined by Hendrickx and Mateus [22], and shared by MPM-Pv-1805.1 include an almost straight distal profile of the tooth, transversal and short marginal undulations on the crown, denticles with strongly developed interdenticular sulci, distal denticles with an apex pointing towards the tip (although the mesial denticles in MPM-Pv-1805.1 are markedly more hooked than in the abelisaurid described by Hendrickx et al [18]), a DSDI (denticle size density index) close to one, an irregular enamel texture, and the presence of apically pointed denticles on the distal carina. The CBR of this specimen (0.44) is similar to that of other abelisaurids (circa 0.5) described by Hendrickx and Mateus [22]. A ziphodont tooth, with a lenticular cross-section of the crown base, and carinae with hooked mesial denticles (although not as prominently hooked), and elongate distal denticles, is reminiscent of the morphotypes 1 and 2 described by Canale et al. [33], which have abelisaurid affinities.

#### Theropoda (?) indet

MPM-PV-18805.2 (Fig 2D) is a large, longitudinally fragmented and eroded tooth (preserved length: 16 mm; maximum diameter: 9.5 mm) preserving enamel patches attached to the dentine. Although its shape remains unknown, the enamel ornamentation is markedly different from that of other theropod or crocodile teeth. It shows an intricate pattern of markedly large rugosities and grooves (Fig 2D), clearly observed at plain sight. This ornamentation is largely similar to that described as “braided enamel texture” by Hendrickx et al. [18,23] in *Acrocanthosaurus*, and to that described as “veined enamel” in spinosaurids, although the enamel ornamentation is markedly smaller in the mentioned taxa. The owner of this large tooth from the Cerro Fortaleza locality remains unidentified.

### Sauropod remains

Sauropoda Marsh, 1878

Eusauropoda Upchurch, 1998

Titanosauriformes Salgado et al., 1997

#### Titanosauria indet

MPM-PV-18805.3 is an isolate fragmented pencil-like (cylindrical) tooth (Fig 2E and 2F). The preserved fragment is only 9 mm long, and is sub-circular in cross section (largest diameter = 6.5 mm). The enamel is smooth, and there are no marked carinae, as observed in *Nigersaurus* and Diplodocoidea [34]. This fragmented tooth is very similar to those described for titanosaurids, which are generally cylindrical, with nearly parallel margins and lack denticles [e.g. 35]. The cross section varies from circular to elliptical, some being gently D-shaped [e.g. 36]. The large amount of titanosaurid bones present in the Cerro Fortaleza Formation suggests the tooth described here belongs to this clade. The slightly compressed section suggests the fragment corresponds to a distal portion of the crown.

### Ankylosaur remains

Thyreophora Nopcsa, 1915

Ankylosauria Osborn, 1923

Nodosauroidea Marsh 1890

#### Nodosauridae indet

MPM-PV-18805.4 (Fig 2G–2I) is a single isolated tooth, consisting of a crown missing its apex and root. The crown is labio-lingually compressed, leaf-like tooth, with few denticles along the mesial and distal carinae. Its total preserved height is 8.5 mm, the maximum width at the base is 3.5 mm. The lingual and labial surfaces are smooth and swollen around the base. There is a cingulum on the lingual side similar to that observed in nodosaurids. The mesial and distal carinae bear a series of approximately 6 or 7 large and irregular in size denticles, although the complete number is unknown because the apex is eroded. There is a constriction just below the crown. The tooth lacks the outer enamel -a common condition in shed nodosaurid teeth from the Late Cretaceous of North America [e.g. 37]. Nodosaurid characters present in this tooth include a well-developed cingulum at the base of the crown [38], and the root constriction below the root-crown contact [39]. There are no clear wear marks along the faces of the crown as in ankylosaurids, and probably the wear pattern in MPM-PV-18805.4 was tooth-to-tooth only on the top of the crowns as in polacanthids and nodosaurids [39]. In ankylosaurids wear facets develop on the crown faces rather than apically across crowns [40]. Also, the presence of a cingulum is more common among nodosaurids than in ankylosaurids [41]. MPM-PV-18805.4 is highly similar to the teeth of *Antarctopelta oliveroi* [42].

#### Ankylosauria indet

**Fig 3:**
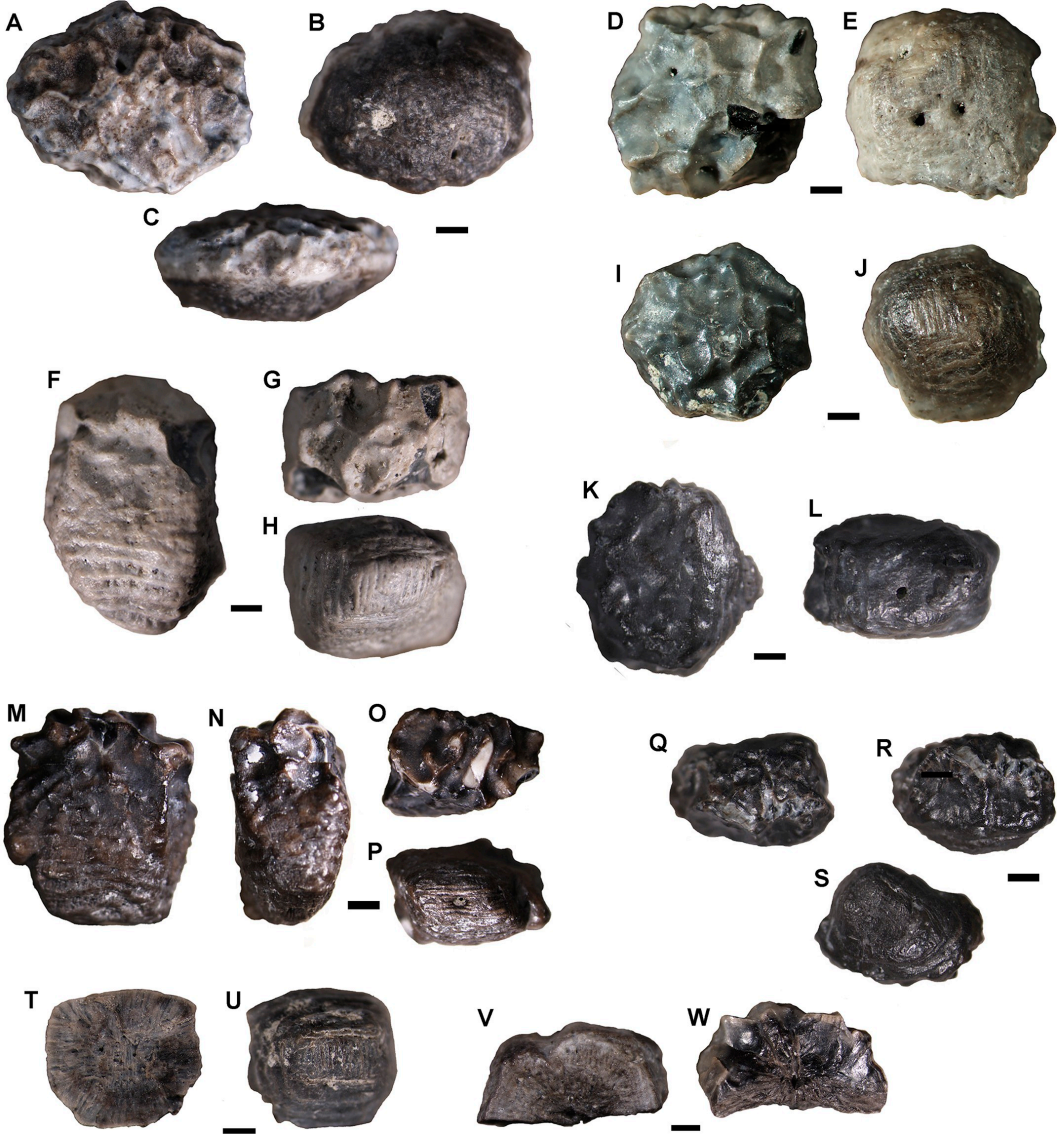
Ankylosaur interstitial ossicles (osteoderms) in superficial, internal and lateral views. MPM-PV-18805.14 (A-C), MPM-PV-18805.15 (D-E), MPM-PV-18805.16 (F-H), MPM-PV-18805.17 (I-J), MPM-PV-18805.18 (K-L), MPM-PV-18805.19 (M-P), MPM-PV-18805.20 (Q-S), MPM-PV-18805.21 (T-U), MPM-PV-18805.22 (V-W). Scale bars = 1mm.

MPM-PV-18805.14–22; small-sized isolated osteoderms (Fig 3). These specimens correspond to interstitial armor ossicles (ossicles that fill the interstitial spaces between larger osteoderms, particularly the ventral side and limbs [see 43–45]. In general terms, these ossicles resemble those reported for the Early Cretaceous Australian nodosaur *Kunbarrasaurus ieversi*, which is considered a small sized ankylosaur, and for *Antarctopelta oliveroi*, which is a possible nodosaurid ankylosaur from Antarctica [46]. Their maximum diameter is approximately 6 mm. Different from other taxa (e.g. *Antarctopelta oliveroi*), the ossicles reported here are heterogeneous regarding their general morphology. Some are oblate spheroid-shaped elements, with a heptagonal contour in superficial view (Fig 3A–3C, 3I and 3J). Others are strongly narrow and tall, having a roughly rectangular outline (Fig 3F–3H and 3M–3P). The deep (= ventral, basal, or internal) surface can be identified by its particular texture, with straight fibers that cross orthogonally, giving a distinct, interwoven texture to the surface (Fig 3H and 3J). This surface bears 1 or 2 neurovascular foramina, which connect with canals internally as shown by fractures and thin sections (Fig 3E and 3W). These features are commonly recorded in the osteoderms deep surface of several other vertebrates, particularly in non-avian dinosaur ossicles [46–51]. The superficial (= dorsal or external) surface is rugose and exhibits several irregular depressions bounded by sharp ridges (Fig 3A, 3D and 3O). A similar pattern has been recorded in the superficial surface of *Antarctopelta oliveroi* ossicles [51].

**Fig 4:**
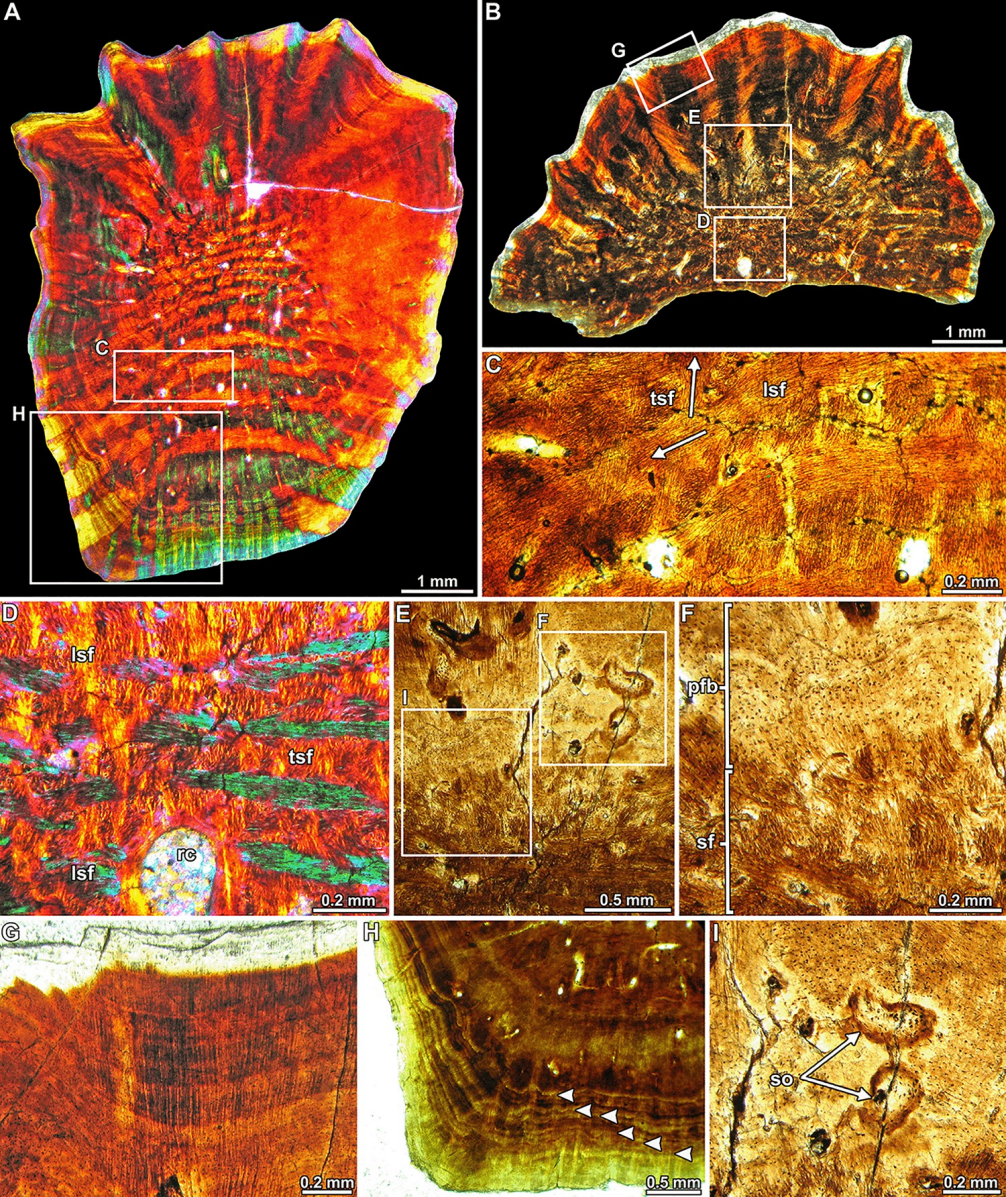
Bone histology of ankylosaur interstitial ossicles. (A-B) Complete sections of elements MPM-PV-18805.19 (A) and MPM-PV-18805.22 (B). In both images the superficial surface is oriented toward the top of the figure. The deep cortex of MPM-PV-18805.v-w is not preserved. The complex pattern of mineralized structural fiber bundles is clearly observed in the inner core and deep cortex of MPM-PV-18805.m-p. (C-D) Primary bone tissue composed by intercrossed bundles of mineralized structural fibers. White arrows in (C) signal the orientation of the fiber bundles. (E, F) General view (E) and detail (F) of the transition between the inner core (formed by structural fibers) and the external cortex (mostly formed by parallel fibered bone). (G) Detail of the outer cortex at the superficial portion of the ossicles. Note the profuse abundance of mineralized collagenous fibers. (H) Lines of arrested growth in the deep cortex (white arrowheads). (I) Detail of secondary osteons. Pictures have been taken under normal transmitted light (C, E-I), cross-polarized light (B) and cross-polarized light with a lambda filter (A and D). The epoxy resin layer that surrounds the elements in (A) and (B) has been digitally erased to enhance visibility. Abbreviations: lsf, longitudinally sectioned structural fiber bundles; pfb, parallel fibered bone; sf, structural fibers; tsf, transversally sectioned structural fiber bundles; rc, resorption cavity; so, secondary osteons.

Two of the interstitial ossicles have been sectioned for histological analysis. The sections were performed in a plane parallel to the superficial/deep axis. The elements are almost entirely composed of compact primary bone tissue, with some vascular spaces located in the inner core (i.e. medullary region) (Fig 4A and 4B). This bone tissue is mainly composed of closely packed bundles of mineralized collagen fibers (i.e. structural fibers), which exhibit a complex and highly ordered spatial organization. In this regard, three systems of fiber bundles are distinguished: one vertical (i.e., parallel to the superficial/deep axis) and two horizontals (i.e., perpendicular to the superficial/deep axis) (Fig 4C and 4D). The horizontal systems are arranged roughly perpendicular to one another. The horizontal bundles become more obliquely oriented toward the external surface in the marginal areas of the ossicles. The bundles located in the inner core are comparatively narrower than those observed in the deep, marginal, and superficial areas. The patterns of intercrossed bundles of structural fibers is distinct between the inner core and the deep cortex, being much more diffuse in the marginal cortex and almost inexistent in the superficial cortex. In this regard, there is an abrupt change in the microstructure at the boundary between the medullary region and the inner portion of the superficial cortex. In this area, the well-defined pattern of intercrossed bundles of structural fibers abruptly changes to an avascular matrix formed by parallel fibered bone, which predominates in the superficial cortex (Fig 4E and 4F). Densely grouped mineralized collagenous fibers predominate in the superficial cortex (Fig 4G). The continuity between these fibers and the structural fiber bundles of the inner core is difficult to assess with confidence. A stratified pattern originated by the presence of cyclical growth marks (i.e., lines of arrested growth) is distinct in the outer portion of the superficial marginal and deep cortices (Fig 4H). Secondary remodeling is only evident for the presence of few resorption cavities and small Haversian systems scattered in the inner core and superficial cortex (Fig 4I).

Millimeter-sized ossicles are known in adult ankylosaurid specimens from the Late Cretaceous of North America [44] and in the nodosaurids *Kunbarrasaurus ieversi* [52,53], *Borealopelta markmitchelli* [54: Fig 1], and *Antarctopelta oliveroi* [46,51]. The oblate spheroid shape and size (approximately 6 mm in diameter) of the ossicles from the Cerro Fortaleza Formation are markedly similar to those described in *Kunbarrasaurus ieversi* by Molnar [52], including the dorsal superficial ornamentation formed by ridges and valleys (APC pers. obs.). A similar pattern has also been observed in the superficial surface of *Antarctopelta oliveroi* ossicles [51]. Regarding the histology of the Cerro Fortaleza Formation ossicles, it is strongly similar to that described for the ossicles of *Antarctopelta oliveroi* [46,51]. The only important difference is the predominance of parallel fibered bone in the specimens here analyzed.

Dermal ossicles possibly formed an extensive basement underlying the epidermal scales of ankylosaurs [44]. Unlike larger osteoderms, interstitial ossicles show no consistent differences among ankylosaur groups, at least among highly nested nodosaurids and ankylosaurids [50]. Thus, it is not possible to assign the specimens to a clade less inclusive than Ankylosauria. Despite its taxonomical uncertainties, this report not only increases the vertebrate diversity of the Cerro Fortaleza Formation, but also represents the first record of ankylosaur interstitial ossicles for South America.

### Peirosaurid remains

Crocodyliformes Hay, 1930 (sensu Benton and Clark, 1988)

Mesoeucrocodylia Whetstone and Whybrow, 1983 (sensu Benton and Clark, 1988)

Notosuchia Gasparini, 1971 (sensu Sereno et al., 2001)

Peirosauridae Gasparini, 1982 (sensu Gasparini et al., 1991)

**Fig 5:**
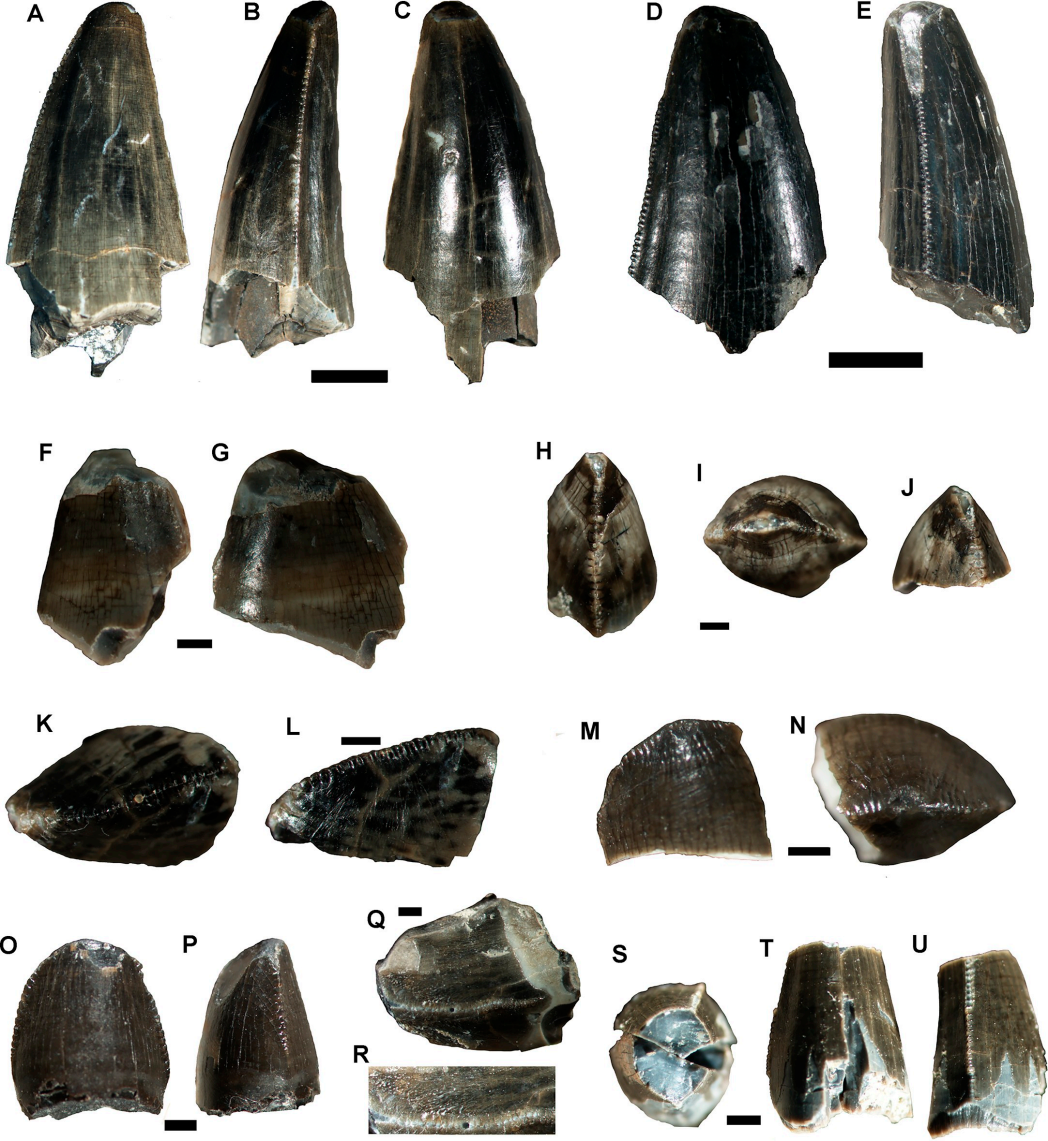
Peirosaurid teeth. MPM-PV-18805.5 complete crown in lingual (A), mesial (B) and labial (C) views. MPM-PV-18805.6 complete crown in labial (D) and distal (E) views. MPM-PV-18805.7 fragmented crown in mesial (F) and lingual (G) views. MPM-PV-18805.8 almost complete crown in mesial or distal (H), apical (I) and mesial or distal (J) views. MPM-PV-18805.9 fragmented crown in mesial or distal (K) and labial (L) views. MPM-PV-18805.10, fragmented crown in labial (M) and apical (N) views. MPM-PV-18805.11, complete crown in lingual (O) and mesial (P) views. MPM-PV-18805.12, fragmented crown in mesial or distal (Q) and detail of the denticles (R). MPM-PV-18805.13, crown missing the tip in apical (S), lingual (T) and distal (U) views. Scale bars (A-E) = 5 mm; (F-U) = 1 mm.

MPM-PV-18805.5–13 comprise nine tooth crowns missing the roots, some of them fragmented (Fig 5). All the teeth correspond to isolated “true” ziphodont teeth [sensu 27]. These are crowns in which the medio-distal diameter is larger than the labio-lingual, and with finely denticulated carinae. The carinae have a continuous series of true isomorphic denticles perpendicular to the margin of the tooth [sensu 55]. These denticles are isolated and separated by interdenticular grooves. The preserved teeth comprise crowns of conical, spatulate, and globose morphology [sensu 28].

The teeth are assigned to Peirosauridae based on their general morphology, which is reminiscent of that observed in other taxa [56–63] in having conical, spatulate and globular tooth crowns, with subcircular or oval cross section, slightly convex faces, finely serrated carinae, and a poorly marked constriction between the crown and the root [64,65] (Fig 5). In addition, peirosaurids are a frequent component of continental Cretaceous vertebrate faunas of southwestern Gondwana [63,66–68]. Within the sample of peirosaurid teeth, four morphotypes are recognized.

#### Morphotype I

**Fig 6:**
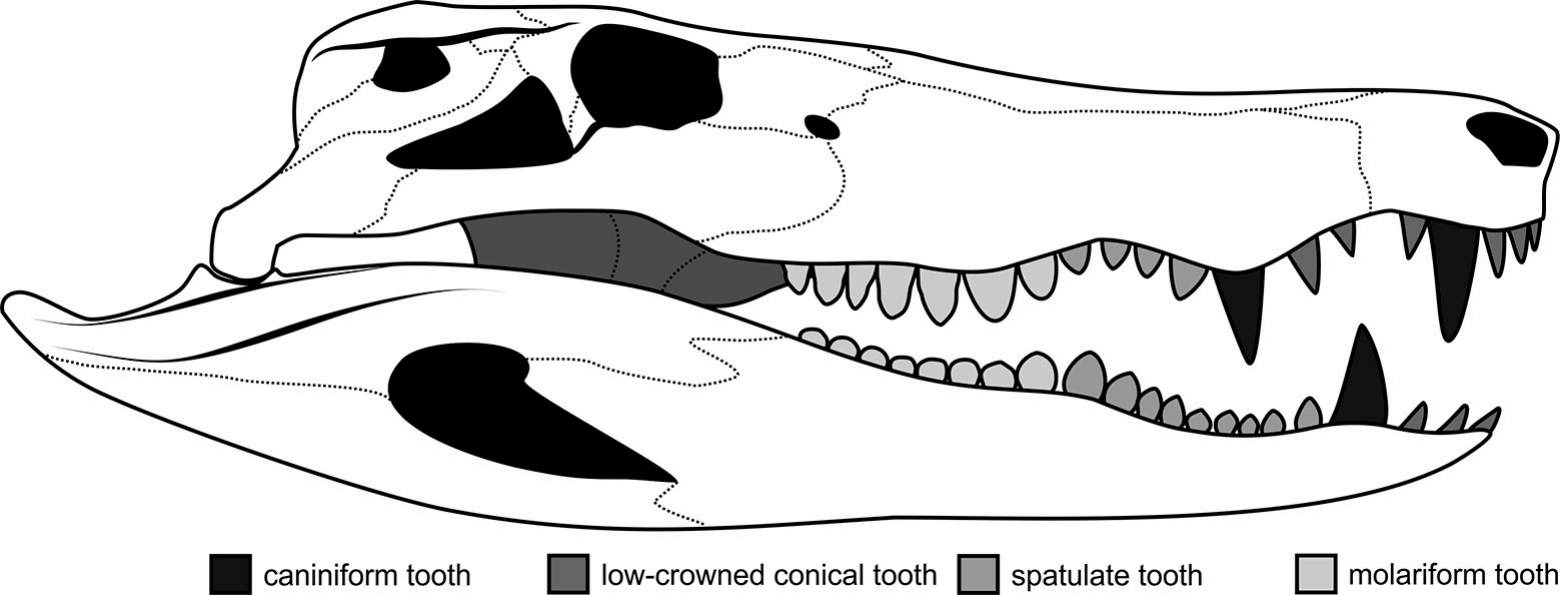
Scheme of a peirosaurid skull showing heterodont teeth and the probable location of morphotypes I to IV. Abbreviations: 1, caniniform tooth; 2, low-crowned conical tooth; 3, spatulate tooth; 4, molariform tooth.

Mophotype I correspond to tall and conical crowns, with a subcircular cross-section. In the peirosaurid skull, this morphology is present in the anteriormost teeth (Fig 6).

MPM-PV-18805.5 (Fig 5A–5C) corresponds to a tooth with most of the crown preserved. It is conical (the base is subcircular in cross-section), apico-basally tall, with a slight constriction at the base, and well-marked mesial and distal carinae. The tooth has a slight lingual curvature distally, a labial face markedly convex, and a lingual face slightly concave. These faces are smooth and are separated by the medial and distal carinae, which bear small denticles. The lingual side of the crown bears a central convexity with a shallow groove mesially, adjacent to the denticulated mesial carina. The denticulated mesial and distal carinae extend from the base to the apex of the crown, with small denticles distributed regularly and similar in size and morphology. The denticles have an external rounded keel and are separated by interdenticular grooves. The apex of the crown has a semicircular wear facet, apical wear [sensu 69], slightly displaced to the mesiolabially side.

MPM-PV-18805.6 (Fig 5D and 5E) is a conical tooth preserving an almost complete crown. It is apico-basally tall and with no constriction at the base, which is subcircular to oval in cross-section. The tooth has a slight lingual curvature, with the labial face strongly convex, and the lingual face slightly concave to straight. Both surfaces are smooth. The labial side bears a shallow groove distally, adjacent to the denticulated carina, and well-marked in the base. The carinae on the mesial and distal edges are serrated with marked denticles. These denticles are small, isomorphic, and regularly distributed, with an external rounded keel and separated by interdenticular grooves. The crown has an apical wear facet extended over the distal carina (apico-carinal wear).

MPM-PV-18805.13 **(**Fig 5S–5U) is a partially preserved conical, and apico-basally tall crown with a circular base in cross-section. The labial face is strongly convex, and the lingual face is slightly concave. They lack the grooves adjacent to the carina mesially and distally. Both faces are smooth and are separated by well-marked serrated carinae (the mesial carina is better preserved). The denticles are small, individual, and separated by interdenticular grooves. The denticle size varies at the base of the mesial carina, where small denticles intercalate sets of one or two larger ones. Denticle external keels are rounded.

#### Morphotype II

Morphotype II corresponds to low and sub-globose conical crowns, subcircular in cross-section, with well-defined constriction between crown and root. In the peirosaurid skull, this morphology is present in anteriormost teeth (Fig 6).

MPM-PV-18805.7 (Fig 5F and 5G) is a fragmented crown, missing most of the apex and basal region. It is possible, however, to determine that it is an apico-basally low crown with conical outline, slightly curved distally. The distal carina has poorly preserved small denticles.

MPM-PV-18805.11 (Fig 5O and 5P) is an almost complete apico-basally low crown, slightly conical and globose, with labial face strongly convex and lingual face slightly convex to straight. Both faces are smooth, separated by well-marked and serrated carinae, and lack the grooves mesially and distally adjacent to the carina. The denticles of the carina are small, distributed regularly, and have well-defined interdenticular grooves. The denticles at the base and the apex are smaller. The external keel of the denticles is apically curved, with interdenticular grooves slightly curve ventrally towards the central region of the crown. The base of the crown is subcircular in cross-section, and has a constriction (which is more marked mesio-distally) that separates the crown from the root. The crown has an elliptical apical wear facet that extends on the labial face.

#### Morphotype III

Morphotype III corresponds to low, labio-lingually compressed crowns, which are oval in cross-section, and have a spatulate outline in lateral view. In the peirosaurid skull, this morphology is present in post-caniniform teeth (Fig 6).

MPM-PV-18805.9 (Fig 5K and 5L) represents half of an apico-basally low and spatulate crown, slightly labiolingually compressed, and oval in cross-section at the base. The labial face is convex and the lingual face is slightly straight, both separated by serrated carinae and lacking grooves adjacent to the carina. The denticles are smaller at the base, regularly distributed and separated by marked interdenticular grooves. The external keel of the denticles is rounded. This crown lacks wear facets.

MPM-PV-18805.10 (Fig 5M and 5N) is a fragmented apico-basally low crown, spatulate in lateral view, and labiolingually compressed. There is a constriction between crown and root, well-marked mesiodistally. The labial face is convex and the lingual face is slightly concave, both separated by well-marked and serrated carinae. The apex has apico-basally extending striations on the labial and lingual faces. The denticles in both carinae are individual (separated by well-defined interdenticular grooves), similar in size, and regularly distributed. The apical interdenticular grooves match the distribution of the above mentioned striations. The external keel of the denticles is rounded. This crown lacks wear facets.

MPM-PV-18805.12 (Fig 5Q and 5R) is a partially preserved apico-basally low and spatulate crown, missing the apex. The base is sub-oval in cross-section. The labial face is convex and the lingual face is slightly concave. Both faces are separated by poorly marked and badly preserved serrated carinae. Labial and lingual faces have irregular apico-basally striations that converge in the carinae, but lacks marginal grooves. The denticles are small and individual, separated from one another by an interdenticular groove. The denticles are regularly distributed, and their sizes vary along the length of the carina; small denticles are intercalated between two larger denticles. Near the base, the preserved (mesial or distal?) carina has a slightly apicobasal undulation, with a small convexity towards the lingual face. This convexity bears relatively larger denticles, whereas the denticles are smaller near the base. On the labial and lingual faces, there are small striations mostly concentrated at the apical region, which are not related to the denticles nor with the interdenticular grooves. The external keel of the denticles is rounded.

#### Morphotype IV

Morphotype IV corresponds to low and globose crowns, molariform type, which are subcircular in cross-section. In the peirosaurid skull, this morphology is present in posteriormost teeth (Fig 6).

MPM-PV-18805.8 (Fig 5H–5J) is an almost complete, apico-basally low crown, globose, and sub-circular in cross-section. Labial and lingual faces are convex and separated by a well-defined and serrated carina. Both faces have small apicobasal striations, well-marked apically. The lingual and labial faces bear a central convexity bordered by a pair of mesial and distal subtle grooves. The denticles are regularly distributed, with smaller elements both apically and basally. Each individual denticle has a rounded external keel, separated from one another by marked interdenticular grooves. In this crown, a small apical wear is present.

## Discussion

### Cretaceous archosaurian record of Cerro Fortaleza Formation

Isolated teeth are commonly found in Mesozoic rocks, the tetrapod faunas of which were dominated by polyphyodont taxa that continually replaced their functional dentitions [21 and references therein]. In many fossil bearing beds, dinosaur and crocodyliform teeth are more common than other well preserved skeletal remains [e.g. 70]. This responds in part to the large number of teeth on their jaws, and also to the fact that most dinosaurs and crocodyliforms had an almost continual supply of teeth that could be shed into the local environment [e.g. 19,21,27,71–74]. However, until now, this was not the case of the Cerro Fortaleza Formation, where isolated teeth are scarce. The identification of the teeth from the micro-remains site studied here, allowed to determine the presence of certain taxa (such as theropod and ankylosaur—probably nodosaur—dinosaurs and peirosaurid crocodyliforms), whose skeletal remains are not yet recorded. The faunal assemblage of dinosaurs is, in part, taxonomically similar to that recently recovered in the Chorrillo Formation [9,10], which is dominated by ornithischians.

The only reports of theropods in the Cerro Fortaleza Formation correspond to the megaraptorid *Orkoraptor burkei* (“Pari Aike” Formation in [6], found in rocks outcropping south Lago Viedma), and the probable [see 75] abelisauroid *Austrocheirus isasii* [12], although only the former has preserved teeth. The theropod tooth MPM-PV-18805.1 from the Cerro Fortaleza locality differs in shape from those of *Orkoraptor burkei* (the crowns of which have a 8-shaped cross section and a curved distal margin) and, more importantly, in the presence of a serrated mesial carina (not serrated in *Orkoraptor burkei*). Remains assignable to Megaraptoridae, Abelisauroidea, and Unenlagiidae were found in the Chorrillo Formation [9], although only one tooth was described (MACN-Pv 19066) and assigned to a megaraptorid. Furthermore, the only record of theropod remains from the Cerro Fortaleza locality corresponds to a partial abelisauroid metatarsus reported recently [17]. Altogether, this suggests that the new tooth probably corresponds to the clade, supporting the presence of mid to large-sized abelisaurids in the Late Cretaceous of southern Patagonia. As for the sauropods, titanosaur remains are commonly found in the Cerro Fortaleza Formation, including *Puertasaurus reuili* [14] and *Dreadnoughtus schrani* [11], which are among the largest-sized sauropods, together with *Nullotitan glaciaris* from the Chorrillo Formation [9]. The fragmented specimen described here, is a pencil‐like tooth with a subcylindrical crown (at least at the preserved section of the tooth), smooth enamel and lacking carinae, all traits observed in the Diplodocoidea [e.g. 76], rebbachisaurids, and highly nested titanosauriforms [77, and references therein]. The lack of the apical region of the tooth described here prevents further comparisons at this point.

The ornithischian record from Cerro Fortaleza Formation is restricted to the elasmarian ornithopod *Talenkauen santacrucensis* [13] and the ankylosaur remains described here from the type locality. From the Chorrillo Formation, the ornithischian fauna includes hadrosaurian remains, the elasmarian ornithopod *Isasicursor santacrucensis*, and an indeterminate ankylosaur. The later constitutes the southernmost record of the clade in South America and together with the specimen from Cerro Fortaleza confirm a geographical link with the ankylosaur record from Antarctica. The ankylosaur fossil record remains scarce in South America, and to date comes only from rocks of Campanian-Maastrichtian age. This is true for other Gondwanan areas, although better preserved specimens have been recovered in Antarctica and Australia [42,53,78]. In Argentina, there are no skull remains of ankylosaurs so far, and the most complete specimen corresponds to the Antarctic taxon *Antarctopelta oliveroi* [42]. The tooth recovered from the Cerro Fortaleza locality probably belongs to a nodosaurid ankylosaur, the same probable affinity of the other specimens collected in Argentina. The first record of ankylosaur remains in the country corresponds to a femur, large osteoderms, and one tooth from the Allen Formation (Campanian-Maastrichtian), northern Patagonia [79,80]. Later, large-sized armor osteoderms were recovered from the Puerto Yeruá Formation (Late Cretaceous?) in Entre Ríos Province [81], La Colonia Formation (Campanian-Maastrichtian) in Chubut Province [82], and more recently from the Allen Formation (Campanian-Maatrichtian), Río Negro Province [83] and the Chorrillo Formation, Santa Cruz Province (Rozadilla et al 2021). None of the above-mentioned materials correspond to small-sized ossicles as those from Cerro Fortaleza Formation. Millimeter-sized ossicles are known in adult ankylosaurid specimens from the Late Cretaceous of North America [44], and in the nodosaurids *Kunbarrasaurus ieversi* [52,53] and *Borealopelta markmitchelli* [54, Fig 1]. However, the osteoderm histological analysis is insufficient to discriminate between ankylosaur families.

The ankylosaur *Antarctopelta oliveroi* [42] was found in the James Ross Island -Antarctic Peninsula- in rocks of Campanian age. At that time, the Antarctic Peninsula was connected to South America, allowing faunistic interchange between both continents, with the nodosaurids probably arriving to South America around the late Campanian, through Central America [e.g. 10,81]. The dinosaur assemblages including ankylosaur remains from Chorrillo [9,10] and Cerro Fortaleza formations are the first records filling the gap between Antarctica and North Patagonia, supporting an ankylosaurid common fauna.

Serrated ziphodont teeth are common within Crocodyliformes [e.g. 27,84,85]. In the Late Cretaceous of Argentina, such teeth have been recorded in Baurusuchidae [86,87] and Peirosauridae [88], the latter being the most common and diverse crocodyliforms during this period [e.g. 62,87,89–92]. In addition, baursuchid teeth are strongly compressed labiolaterally and distally recurved [e.g. 93], unlike those of peirosaurids. The peirosaurid teeth presented here exhibit heterodonty, and correspond to the most austral record of the clade so far. The previous record of peirosaurids in southern Patagonia include *Colhuehuapisuchus lunai* from Chubut Province [63], although none of the teeth described here exhibit autapomorphies of this taxon. This suggests the presence of at least two different taxa in Southern Patagonia. More comprehensive explorations, and more complete crocodyliform specimens from the Cerro Fortaleza locality and Formation will shed some light on our understanding of the diversity of the clade at these latitudes.

Considering the morphological variation observed along the tooth row (i.e., anterior teeth have conical, tall, and circular cross-sectional crowns, whereas posterior teeth have low, spatulate and globose crowns, which are oval to circular in cross-section) in extant and many extinct crocodyliform taxa [27–29] four morphotypes were identified. Morphotype I corresponds to an anterior caniniform tooth, morphotype II corresponds to low conical teeth anteriorly positioned, morphotype III corresponds to a tooth of an intermediate position, and morphotype IV correspond to molariform teeth, i.e., the type of tooth most posteriorly positioned in the peirosaurid jaws (Fig 6). Strikingly, the denticle morphology observed in MPM-PV-18805.11 (Fig 5O and 5P), corresponding to the morphotype II, is recognized in some teeth of the African peirosaurid *Hamadasuchus rebouli* [27], but not in South American taxa. This suggests that MPM-PV-18805.11 belongs to a taxon with more affinities with African than South American peirosaurids.

### Implications of the faunal association

One of the first steps in studying interactions among extinct organisms and their environments is identifying the taxa that comprised the ecosystem [21]. Dinosaur associations of ankylosaur, ornithopod, theropod, and sauropod remains have been reported globally, based on bones [e.g. 94] or tracks [e.g. 95,96]. In Patagonia in particular, the dinosaur taxonomic richness of some Cretaceous stratigraphic units and sites is considered high [e.g. 97,98]. However, in southern Patagonia, only the Chorrillo and Cerro Fortaleza formations recorded ankylosaur remains [10], and only the later peirosaurid remains. The micro-remains site at Cerro Fortaleza indicates the coeval association of titanosaur sauropods, abelisauroid theropods, ankylosaurs (probably nodosaurs), ornithopods, and peirosaurids. The peirosaurid crocodyliform teeth represent 75% of the sample, suggesting a predominance of crocodyliforms over dinosaurs, which is congruent with the rise of notosuchian diversity during the Late Cretaceous [99]. This statement however, should be supported by taphonomical analyses or further findings in the area. The coexistence of different species of large reptiles is possible if they occupy differ niches, reducing the competition for resources [100]. In terms of other taxonomical components of the ecosystem, the previously reported non-dinosaur fauna of the Cerro Fortaleza locality is diverse, including fishes and turtles. The studied flora in this area indicates a high predominance (75:25) of gymnosperms over angiosperms, and interestingly, these fossil woods also provided evidence for seasonal growth regimens in the region, based on growth rings [4]. This supports a pronounced seasonality caused by rainfall patterns based on sedimentological analyses [8]. Furthermore, the presence of *Zamuneria amyla* (Cycadales) in the Cerro Fortaleza locality indicates humid and warm climate, as extant cycads grow in tropical to subtropical areas [101].

Among the ankylosaur remains from both the Chorrillo and Cerrro Fortaleza formations, particularly the tooth recovered from the later exhibits nodosaurian traits. Interestingly, nodosaurids are found with high frequency in coastal environments. It has been suggested that either this group inhabited a broader range of paleo-environments than ankylosaurids, or even that nodosaurids alone preferred such environments [102,103, contra 104]. In congruence with these hypothesis, the Cerro Fortaleza Formation is characterized by lithified fluvial sands, overbank mud deposits, and paleosols deposited in fluvial, fluvial–palustrine, and coastal plain environments from the northeastern margin of the Austral Basin [1,16,4]. In turn, the continental Chorrillo Formation is part of a rock succession that conforms a late Campanian-early Maastrichtian regressive episode, characterized by braided and meandering fluvial deposits [2,9].

Both, Chorrillo and Cerro Fortaleza formations share a faunal association with a dinosaur diversity composed by large sauropods, abelisaurid and megaraptorid theropods, ornithopods, and ankylosaurs. Differences in faunal composition rely on the hadrosaurids recovered only in Chorrillo Formation, and peirosaurids recorded only in Cerro Fortaleza Formation. So far, this supports both the Campanian (approximately 72 My [16]) and Maastrichtian [16] ages proposed for Cerro Fortaleza Formation that probably correlates with the lower section of the Chorrillo Formation (Campanian-Maastrichtian in age) [10]. However, further discoveries are necessary to better understand the correlation between these two formations.

**Fig 7:**
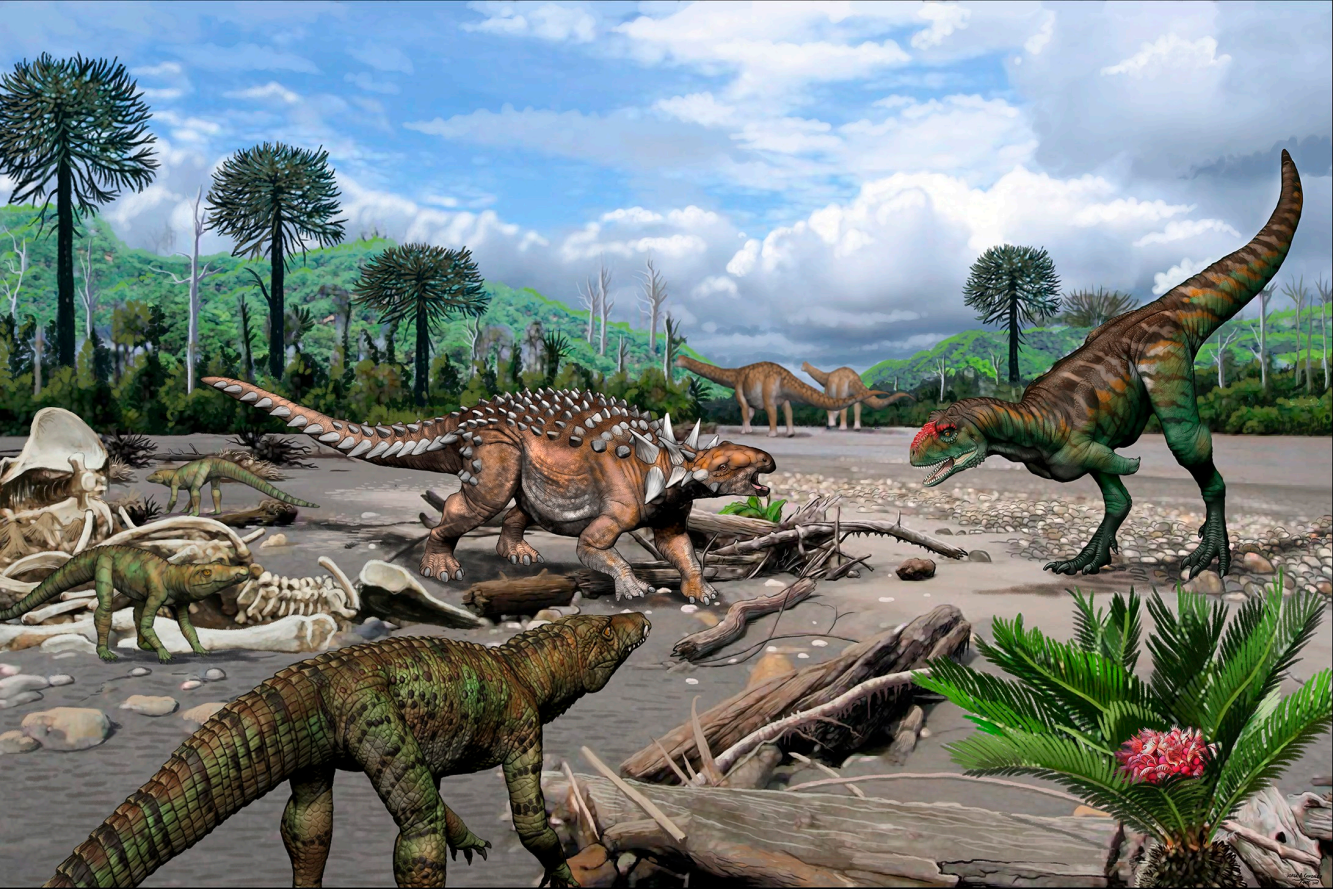
Paleoambiental reconstruction of Cerro Fortaleza locality (Cerro Fortaleza Formation) showing the coeval dinosaurs and peirosaurid notosuchians described in the present study (and right below is *Zamuneria*). Illustration by J. González.

In this panorama, although it is more likely that the deposition of teeth found in the site at Cerro Fortaleza locality was an attritional event, the data provided by this micro-remains site indicates that at least titanosaur sauropods, abelisaurid and megaraptorid theropods, elasmarian ornithopods, nodosaurid ankylosaurs, and a variate number of crocodyliform taxa were part of the same late Cretaceous ecosystem. This paleoenvironment, as indicated by the fossil record, sedimentology, and paleoclimate interpretations for the area, was characterized by a meandering fluvial system converging with a shore landscape, associated with a subtropical forest (Fig 7).

## Conclusions

Here, we report the faunal taxonomic composition of a micro-remains site at Cerro Fortaleza locality (Cerro Fortaleza Formation, Campanian-Maastrichtian) based on isolated teeth and osteoderms, providing insights into the paleobiodiversity of a Late Cretaceous ecosystem in South America. Although scarce, the tooth sample from Cerro Fortaleza locality is taxonomically rich, representing different dinosaur and crocodyliform clades that probably cohabitated the same bioma during the Late Cretaceous. This report enriches the faunal knowledge in a site where other kind of skeletal remains poorly represent these clades (except for the sauropods), or were not previously reported. The archosaur taxonomic composition of the Cerro Fortaleza Formation includes Titanosauria, Theropoda (Abelisauridae and Megaraptoridae), Ornithischia (Ornithopoda and Ankylosauria), and notosuchian crocodyliforms (Peirosauridae), with the later representing the predominant archosaurs in the studied sample. The peirosaurid teeth from Cerro Fortaleza locality are the first recorded at this latitude. The ankylosaur specimens from Cerro Fortaleza and Chorrillo formations, indicate that this group of dinosaurs reached an austral distribution at least since the late Campanian, filling the gap in the fossil record between Antarctica and central-northern Patagonia.
